# Genome-Wide Analysis of MicroRNA-related Single Nucleotide Polymorphisms (SNPs) in Mouse Genome

**DOI:** 10.1038/s41598-020-62588-6

**Published:** 2020-04-01

**Authors:** Gideon Omariba, Fuyi Xu, Maochun Wang, Kai Li, Yuxun Zhou, Junhua Xiao

**Affiliations:** 1 College of Chemistry, Chemical Engineering, and Biotechnology, Donghua University, Shanghai, 201620 China; 2 Department of Genetics, Genomics and Informatics, University of Tennessee Health Science Center, Memphis, TN 38163 United States

**Keywords:** Genetic databases, Mutation

## Abstract

MicroRNAs are widely referred to as gene expression regulators for different diseases. The integration between single nucleotide polymorphisms (SNPs) and miRNAs has been associated with both human and animal diseases. In order to gain new insights on the effects of SNPs on miRNA and their related sequences, we steadily characterized a whole mouse genome miRNA related SNPs, analyzed their effects on the miRNA structural stability and target alteration. In this study, we collected 73643859 SNPs across the mouse genome, analyzed 1187 pre-miRNAs and 2027 mature miRNAs. Upon mapping the SNPs, 1700 of them were identified in 702 pre-miRNAs and 609 SNPs in mature miRNAs. We also discovered that SNP densities of the pre-miRNA and mature miRNAs are lower than the adjacent flanking regions. Also the flanking regions far away from miRNAs appeared to have higher SNP density. In addition, we also found that transitions were more frequent than transversions in miRNAs. Notably, 841 SNPs could change their corresponding miRNA’s secondary structure from stable to unstable. We also performed target gain and loss analysis of 163 miRNAs and our results showed that few miRNAs remained unchanged and many miRNAs from wild mice gained target site. These results outline the first case of SNP variations in the mouse whole genome scale. Those miRNAs with changes in structure or target could be of interest for further studies.

## Introduction

MicroRNAs (miRNAs) are non-coding RNA molecules with a length of about 18–24 nucleotides^1^. Initially, miRNAs are transcribed from primary (pri-) miRNA transcript by the aid of a microprocessor complex called Drosha, to form the precursor (pre-miRNA) in the nucleus^2^. Then pre-miRNAs are transported to the cytoplasm for more processing into miRNA duplexes. The duplex is then weighed down onto Argonaut (Ago) protein of the RNA induced silencing complex (RISC) producing a mature single stranded miRNA^3,4^. It is estimated that untranslated region (UTR) is the potential region for post transcriptional regulation of mRNA^5^. Currently several miRNAs have been identified in various species including human and mice^6^. It is believed that miRNAs are involved in various biological processes by targeting mRNA induces translational inhibition of target gene^7^. Since the miRNA gene regulatory capacity was discovered^8,9^, researchers have developed mechanisms of finding out the potential of miRNAs as causative and therapeutic agent for diseases like cancer and heart failure^10,11^.

Single nucleotide polymorphism (SNP) is one of the most common genetic variations in a genome^12^. SNPs have been reported to have an important role on gene function and regulation^13^. With the advance of high throughput technology, increasing number of research has revealed that SNPs have profound influence in miRNA function, stability and targeting^14^. The SNPs are commonly identified in the miRNA genes, or in the binding site of mRNA of miRNAs. SNPs in the miRNA binding site are capable of modifying miRNA by generating or removing a miRNA binding site in the target mRNA^15,16^. These SNPs can effectually cause target alterations and gene expression interference^17^. To date, it has been reported by a number studies that SNPs in miRNAs genes or in the target site are involved in complex traits and diseases^18–25^. For example pre miR-146a- SNP (rs2910164) was reported to be associated with the reduced expression of the pre-miR-146a^19,21^. Also the association of mir-499-SNP (rs3746444) with various immunity diseases was reported^22,24,25^. Zhou *et al*., have also identified common polymorphism pre-miRNA associated with cardiomyopathy^23^.

So far, a number of lines of inbred mice with several isolated mutations have been established. This has enriched genetic diversity and alterations giving light to mammalian biology and human disease at large^26,27^. The mouse is one of the potential and extraordinary organisms towards our understanding of human gene function^28^. About 3,587 mouse genotypes, modeling human diseases have been reported, with at least one or more mutated genes. Around 700 inbred mice lines have been developed and are used for genetic mapping studies^29^. The variations in these inbred lines have enabled the identification of genes and variations contributing to complex traits, quantitative trait locus (QTLs) and diseases like cancers, infectious diseases^30^ physiology behavior^31^ and reproduction.

Despite of fierce research done on human genome and other species, very little research has been done on miRNA-related SNPs in mouse genome. In this study, we steadily characterize whole mice genome miRNA-SNPs, analyze their effects on the miRNA secondary structure stability and target alteration (target gain and loss). We discovered that SNP densities of the pre-miRNA and mature miRNAs are lower than the adjacent flanking regions. We also found that, 841 miRNAs could change their secondary structure from stable to unstable. The results also depict a significant change in miRNA target interaction in the miRNA seed region. These results outline the first case of SNP variations in the whole mice genome, which creates a better platform for further analysis and applications on human disease.

## Data collection and characteristics of miRNA with SNPs

In this study, we totally collected 73643859 unique SNPs across the mouse genome from three databases, in which, 68 million SNPs from dbSNP146 of NCBI, 59 million SNPs from MGP(v5), and 3.7 million SNPs from C1SLs (Table 1). There are 1187 pre-miRNAs and 2027 mature miRNAs were deposited in miRBase. After mapping the 7.4 million SNPs onto the mouse pre-miRNAs, 1700 SNPs were identified in 702 pre-miRNAs, with 1–14 SNPs in every pre-miRNA, approximately. We also discovered that out of the 1700 SNPs, 609 of them are located in 4624 mature miRNAs while 173 of them are located in 163 seed regions of the miRNA (Table 1).

**Table 1:** Summary of data collection and miRNA related statistics.

Term	Number
SNPs in dbSNP146	67850467
SNPs in MGP(v5)	59241109
SNPs in C1SLs	3727081
Total SNPs	73643859
Total pre-miRNAs	1187
Total miRNA	2027
pre-miRNA containing SNPs	702
SNPs in pre-miRNA	1700
mature miRNA containing SNPs	464
SNPs in miRNA	609
seed region containing SNPs	163
Numberof SNPs in seed region	175

**Figure 1:**
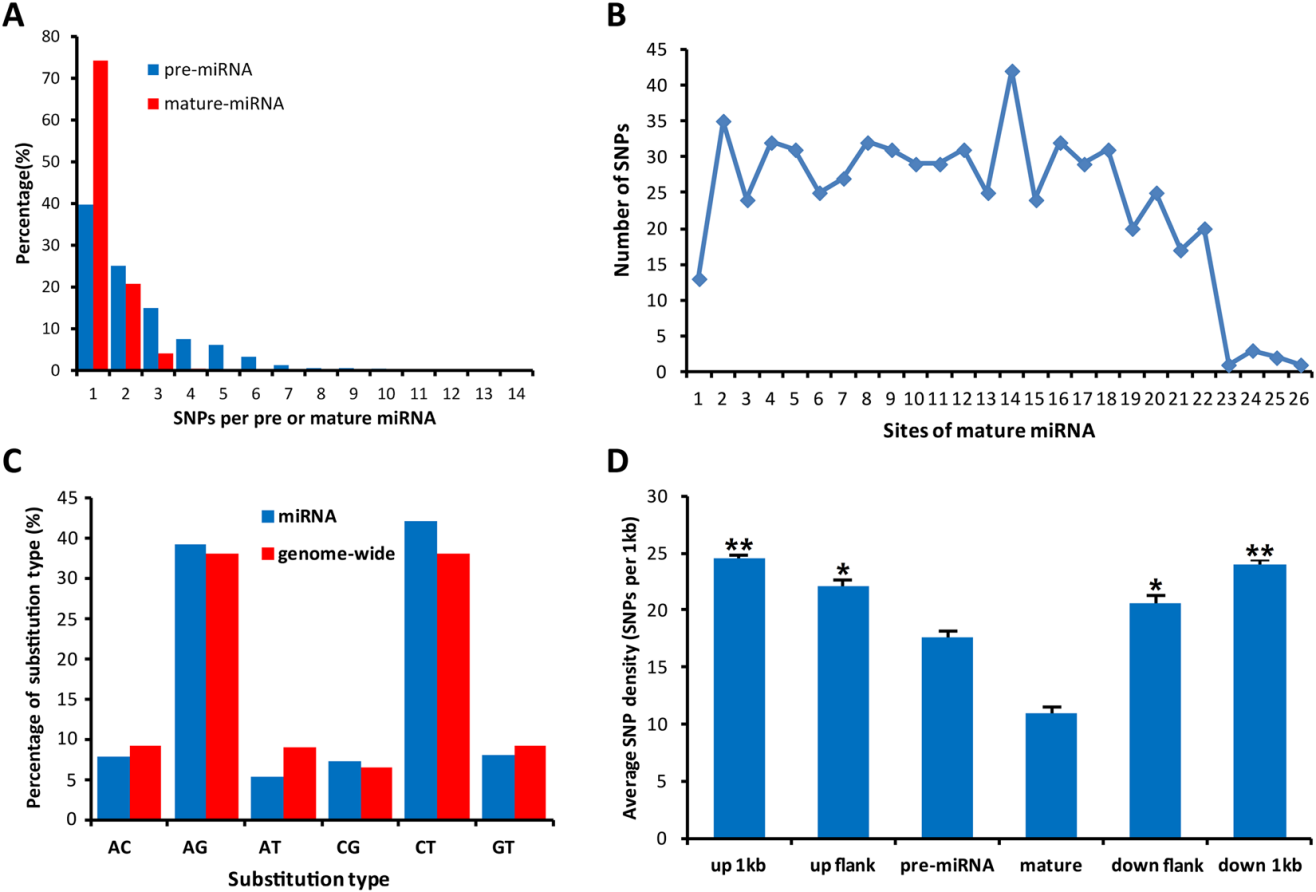
Characteristics of miRNA genes with SNPs. (**A**) Frequency distributions for various SNP numbers in pre-miRNAs and mature miRNAs. (**B**) SNP distribution per base along the mature miRNA. (**C**) Types of substitutions identified in mouse pre-miRNAs. (**D**) SNP density of pre-miRNAs, mature-miRNAs, and their flanking regions. The up or down flanking regions are equal to the pre-miRNAs length, they are in the adjacent location to the corresponding pre-miRNAs. The evaluation of these regional differences was done by T test (* represents p < 0.1 and ** represent p < 0.01).

As shown in Fig. 1A, Among the 702 pre-miRNAs with SNPs, 279 (40%) pre-miRNA genes contain one SNPs, 176 (25%) contain two SNPs, and 105 (15%) have three SNPs. The total number of pre-miRNA genes have more than 3 SNPs are 142 which account for 20% of the pre-miRNAs, with only 3 pre-miRNAs having more than 10 SNPs. The highest number of SNPs is recorded mmu-miR-6238 (14), which is followed by mmu-miR-713 (12) and finally mmu-miR-6339 (11). For mature miRNA, nearly 77% (1565) do not contain any SNPs, while for the 462 mature miRNAs with SNPs, most of them are only contain one (343, 74% or two (96, 21%) SNPs. The rest of them containing 3~6 SNPs only account for 4% (23), with mmu-miR-1931 having the most SNPs (6), followed by mmu-miR- 3473b (5), mmu-miR-3060-3p (4), and mmu-miR-7037-3p(4). We also analyzed the SNP distribution per base along the mature miRNA. Results showed that sites including 1, 3, 6, 13, and 15 especially for site 1 tend to have accumulated less SNPs, while sites 2 and 14 tend to containing more SNPs (Fig. 1B).

Data has shown in mean + SE. We also compared various SNP densities in different regions, characterized them according to their flanking regions and upstream or downstream regions. The results show that mature miRNAs have the lowest SNP density (11 SNPs per kb, SD = 20, SE = 0.45). Pre-miRNAs also shows lower SNPs density with 17.5 SNPs per kb (SD = 21.5, SE = 0.62). Moreover, the flanking regions that are more far away from miRNAs genes tend to have higher SNP density. As a comparison, SNP densities of the pre or mature miRNAs are significantly lower than its adjacent flanking regions (Fig. 1D).

Moreover, we additionally found that transitions (71.3%) were undeniably more continuous than transversions (28.7%) in the miRNAs. When compared with genome wide, transitions in miRNAs are higher than in genome wide (71.3% vs 66.0%). For transition, A-C, A-T, and G-T substitutions are lower in miRNAs especially for A-T (5.3% vs. 9.0%), while C-G substitutions in miRNAs are slightly higher than those in genome wide (7.3% vs. 6%) (Fig. 1C).

## Secondary structural stability of miRNA with SNPs

**Figure 2:**
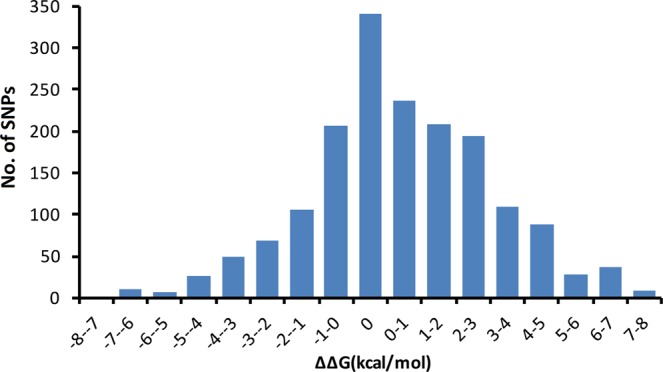
Frequency distribution of energy change (ΔΔG, kcal/mol) for the secondary structure of miRNAs caused by SNPs.

**Figure 3:**
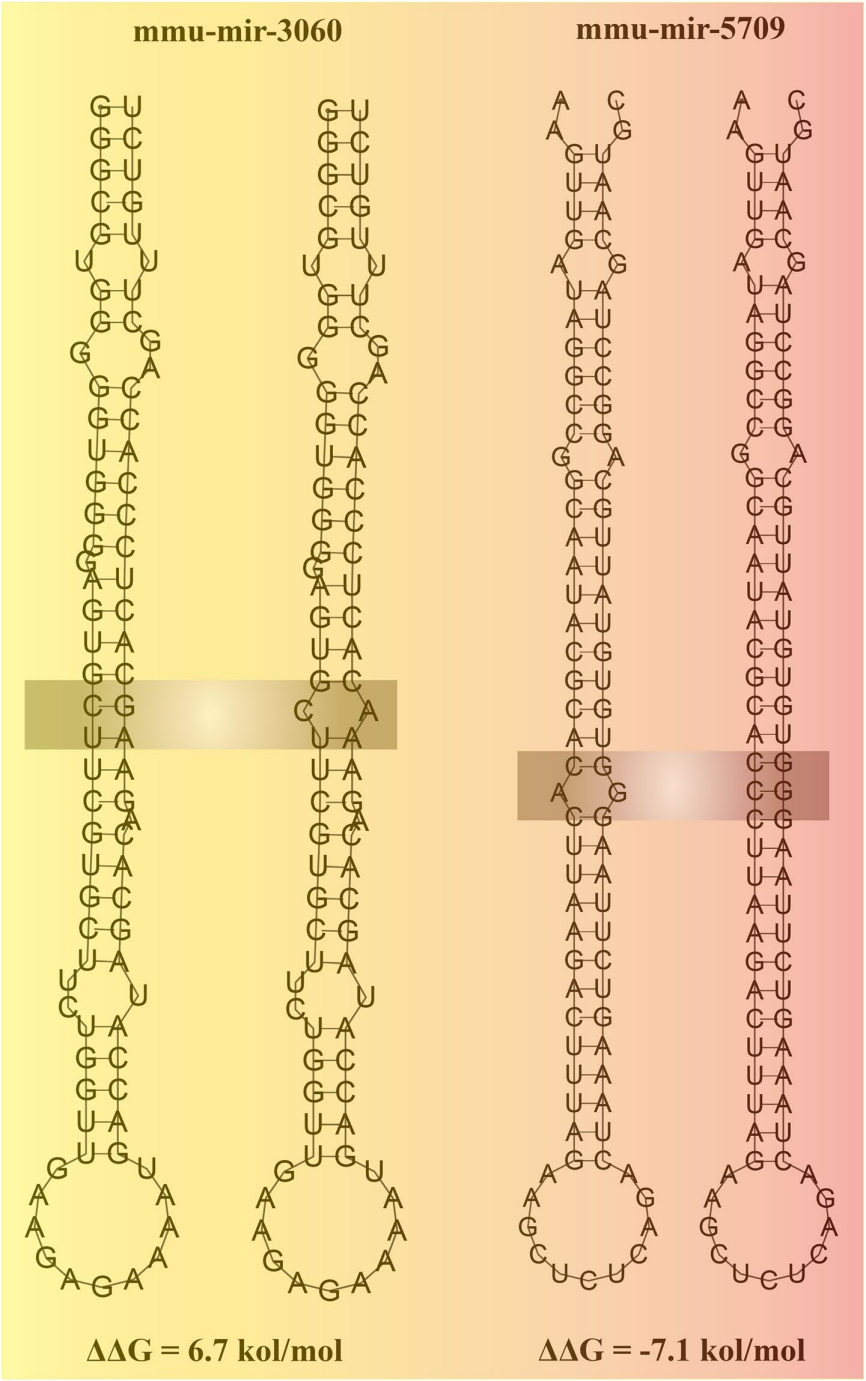
Illustration of the secondary structure of miRNAs with SNPs. For mmu-mir-3060, nucleotide G changed to A causing a small loop structure with a 6.7 kcal/mol MEF change, while mmu-mir-5709 A-to-C SNP in the stem region made its hairpin structure more stable with a −7.1 kcol/mol change.

So as to explore the impact of SNPs in dependability on miRNAs secondary structure, we utilized program RNAfold for secondary structure prediction and looked at the vitality change between wild and mutant type of each miRNAs. As shown in Fig. 2, 1387 SNPs in 626 miRNAs were changed MFE with |ΔΔG| ranging from 0.1 to 7.2 kcal/mol (Supplementary Table 1). The average free energy change caused by SNP on secondary structure is 2.17 kcal/mol4 which is similar with the one in the human genome (2.1 kcal/mol). Previous study showed that the base energy for maintaining the miRNAs secondary structure stability would be 0.3 kcal/mol^32^. We found 841 SNPs in 476 miRNAs could change their secondary structure from stable to unstable according to this criterion (Supplementary Table 1). For example, mmu-miR-3060, where a nucleotide G changed to A, caused a small loop structure with 6.7 kcal/mol MEF change (Fig. 3). On the other hand, 398 SNPs in 294 miRNAs increased the stability of these miRNAs (Supplementary Table 1) such as mmu-miR-5709, where a A-C SNP seems to stabilize the structure of its hairpin more at the stem part with −7.1 kcol/mol MEF change (Fig. 3).

**Figure 4:**
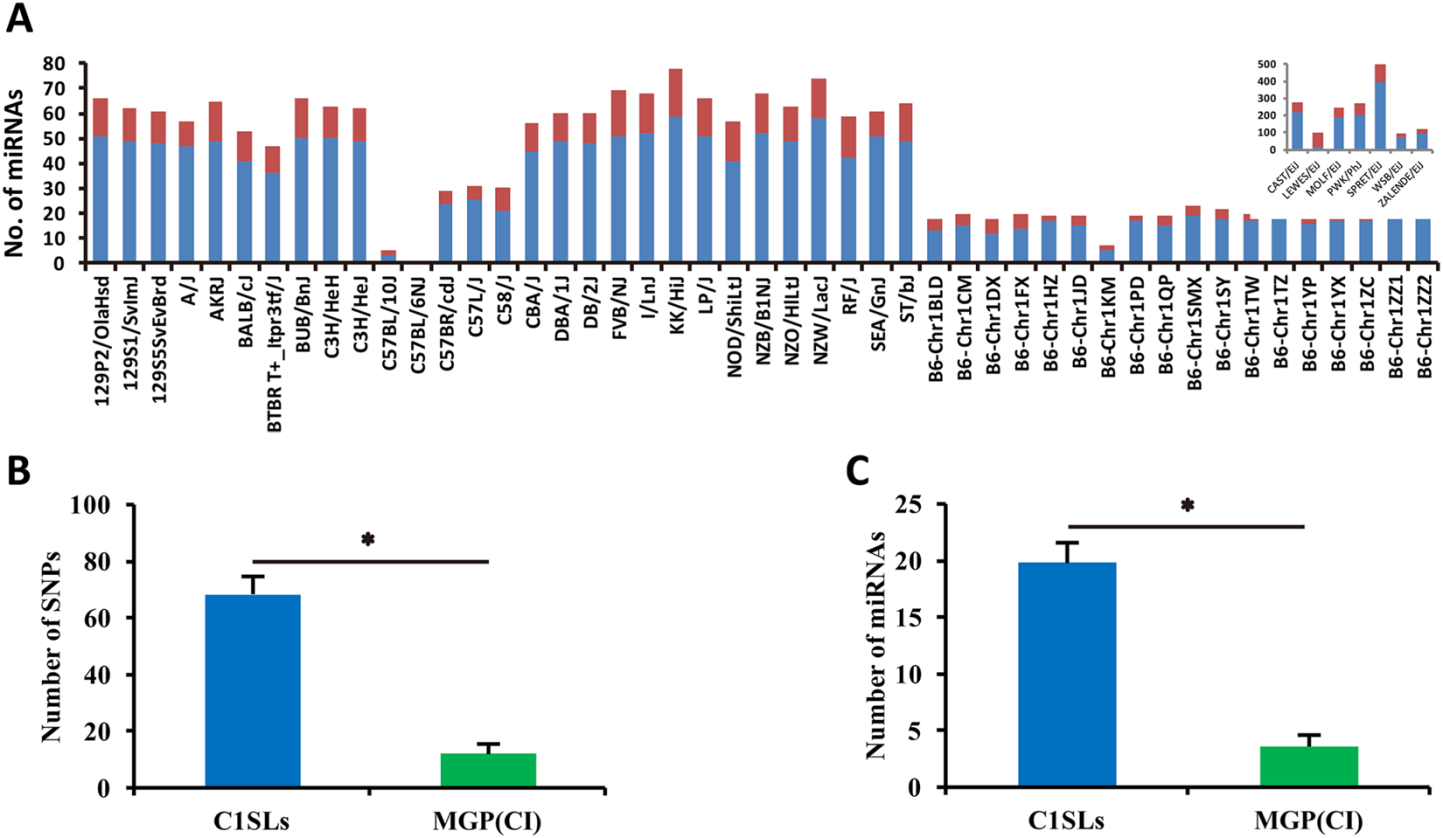
Number of miRNAs containing SNPs in 36 MGP and 18 C1SLs mice. (**A**) The number of miRNAs containing SNPs in 36 MGP and 18 C1SLs. The upper right shows 7 wild-derived strains. Blue bar represents |ΔΔG| > 0.3 kcal/mol of the secondry structure of miRNAs, while red bar represents |ΔΔG| <= 0.3 kcal/mol. Upper right: seven wild-derived mice. (**B**) Shows the number of miRNA SNPs in C1SLs mice and MGP strains. (**C**) Number of miRNAs in C1SLs and MGP mice.

Although the above analysis for miRNAs can evaluate single SNP effect on its secondary structure, it could not reflect the strain-specific miRNA secondary structure change. In other words, for a certain strain, it may contain more than one SNP in one miRNA sequence. Therefore, the secondary structure caused by single SNP or by combining all SNPs would result in to a very different result. We next performed the strain-specific miRNAs secondary structure analysis in 36 MGP and 18 C1SLs mice which we had their exact genotype information. As shown in Fig. 4A, on average, each strain in MGP contain 89 miRNAs (SD = 92.3) genome wide with C57BL/10 J mouse contain only 5 and SPRET/EiJ mouse with 502. While for C1SLs, each line has 19.8 miRNAs (SD = 3.5) in chromosome 1 which is significantly higher than in MGP sequenced 29 classical inbred (CI) strains (excluding seven wild-derived strains) (Fig. 4B,C). Next, we performed the secondary structure analysis, results showed that the overall proportion of ΔΔG > 0.3 kcal/mol for strain-specific was account for 76.4% (SD = 11.6). In addition, we found 9 miRNA with |ΔΔG| over 10 kcol/mol in 24 strains (Table 2). The detailed information for line-specific secondary change was listed in Supplementary Table 2.

**Table 2:** Lists of miRNAs with |ΔΔG| > 10 kcol/mol.

miRNA	Wild MFE	Mutant MFE	ΔΔG	Strain	No. of SNPs
mmu-mir-3060	42.4	27.7	−14.7	BUB/BnJ	4
mmu-mir-3060	42.4	29.9	−12.5	CAST/EiJ	4
mmu-mir-3060	42.4	27.7	−14.7	LEWES/EiJ	4
mmu-mir-3060	42.4	27.7	−14.7	NOD/ShiLtJ	4
mmu-mir-3060	42.4	27.7	−14.7	NZO/HlLtJ	4
mmu-mir-3060	42.4	27.7	−14.7	NZW/LacJ	4
mmu-mir-3060	42.4	27.7	−14.7	ST/bJ	4
mmu-mir-3060	42.4	27.7	−14.7	WSB/EiJ	4
mmu-mir-3470b	47.1	35	−12.1	CAST/EiJ	8
mmu-mir-3470b	47.1	35	−12.1	MOLF/EiJ	8
mmu-mir-3473b	42.5	16.6	−25.9	LEWES/EiJ	6
mmu-mir-3473b	42.5	24.5	−18	PWK/PhJ	3
mmu-mir-3473b	42.5	24.5	−18	SPRET/EiJ	3
mmu-mir-3473b	42.5	16.6	−25.9	WSB/EiJ	6
mmu-mir-3473b	42.5	16.6	−25.9	ZALENDE/EiJ	6
mmu-mir-3473c	41.4	29.5	−11.9	SPRET/EiJ	2
mmu-mir-3473f	69.5	58.1	−11.4	B6-Chr1BLD	3
mmu-mir-3473f	69.5	58.1	−11.4	B6-Chr1CM	3
mmu-mir-3473f	69.5	56.9	−12.6	B6-Chr1HZ	4
mmu-mir-3473f	69.5	58.1	−11.4	B6-Chr1JD	3
mmu-mir-3473f	69.5	58.1	−11.4	B6-Chr1SMX	3
mmu-mir-3473f	69.5	58.1	−11.4	B6-Chr1SY	3
mmu-mir-3473f	69.5	58.1	−11.4	B6-Chr1TW	3
mmu-mir-3473f	69.5	56.9	−12.6	B6-Chr1TZ	4
mmu-mir-3473f	69.5	58.1	−11.4	B6-Chr1YX	3
mmu-mir-3473f	69.5	58.1	−11.4	B6-Chr1ZC	3
mmu-mir-3473f	69.5	58.1	−11.4	B6-Chr1ZZ1	3
mmu-mir-3473f	69.5	58.1	−11.4	B6-Chr1ZZ2	3
mmu-mir-5135	44.1	31.1	−13	MOLF/EiJ	3
mmu-mir-5135	44.1	31.1	−13	PWK/PhJ	3

## Target loss and gain analysis

**Figure 5:**
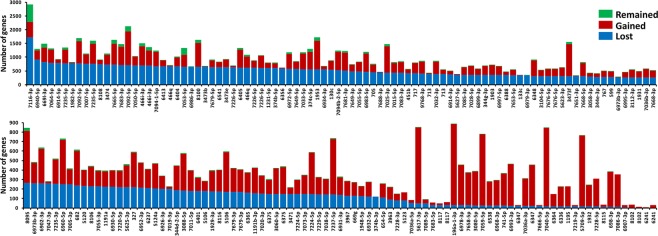
Target alteration analysis of miRNAs in regard with their seed regions. The green bar represents the number of target sites that neither gained nor lost. The red and blue bar represent the number of target sites that gained and lost, respectively.

The miRNA’s target loss and gain is greatly affected by presence of SNPs in the seed regions of the miRNA, thus affecting the miRNA function dramatically. In this study, we obtained our potential miRNA target loss and gain using the comparison difference of wild type targets and SNP- miRNA targets. According to the results, we discovered that few miRNAs remained unchanged, while many of them gained and lost target site respectively (Fig. 5). Interestingly, many miRNAs gained target site happen to be in the wild mice category.

## Discussion

In this study, we steadily characterize a whole mouse genome miRNA SNPs, analyze their effects on miRNA secondary structure stability and target alteration (target gain and loss). Totally, we collected 73643859 SNPs across the mouse genome. When we mapped the SNPs, 1700 SNPs were identified in 702 pre-miRNAs, 609 SNPs were located in mature miRNAs and 173 SNPs in 163 miRNA seed regions. The SNP distribution per base analysis showed different SNP accumulation along the mature miRNA. We discovered that SNP densities of the pre-miRNA and mature miRNAs are lower than the adjacent flanking regions. The flanking regions that are far away from miRNA genes were found to have higher SNP density than the closer ones. We also found that the secondary structure of 476 miRNAs could change from stable to unstable. The results also depict a significant change in miRNA target interaction in the seed regions of the miRNA. In our target gain and loss analysis, results showed that few of the miRNAs remained unchanged. Most of the miRNAs that gained target belong to wild mice category. These results outline the first case of SNP variations in the whole mouse genome, which creates a better platform for further analysis and applications on human disease. The wild type miRNAs that gained target site also create an interesting incite for further studies.

It is worthy to note that most of our findings are consistent with other similar studies done on different species. In our SNP density analysis, we discovered that pre-miRNAs and mature miRNAs have lower SNP densities than the flanking regions which agree with most studies. For instance, a study done by Gong *et al*., on human genome discovered that SNP densities of the flanking regions were higher than SNPs of pre-miRNAs and MIR seed region^33^. In another study, Liu *et al*., found that most research done on rice had indicated that pre-miRNAs contained higher SNP density compared to flanking regions which was contrary to their findings. In their results, they unexpectedly discovered that the SNP densities in the mature miRNAs and pre-miRNAs were higher than SNPs in the flanking regions, which could be as a result of hasty evolutions on miRNAs in specific species of rice^34^. It is also worthy to note that in another cucumber study^35^, they discovered that mature sequences contained less SNP than the binding sites. These divergent results in rice need an in-depth analysis to find out on why some few species have different SNP densities from the rest. We also realized that the far the flanking regions are from miRNA genes, the higher the SNP density they have. Our target loss and gain analysis results showed that most miRNAs either lost or gained targets, which concurs with Gong *et al*.^33^, who discovered that miRNAs SNPs in the seed regions could highly lead to target gain and loss in humans. They also experimentally validated the target loss of miR-124. It is also important to highlight that Harnprasopwat *et al*., found out that an A/G SNP in mature miR-26 could destabilize the secondary structure of pri-miRNA126 and prevent its processing to mature miRNA^36^. On the other hand, SNP G/A at the stem region of miR510 led to mature miRNA production^37^. In our study, we equally agree with others that SNPs could cause unstable hairpin structures in the stem regions hence affect the production of mature miRNAs and affect their stability.

Despite of the mouse being one of the potential and extraordinary organisms towards our understanding of human gene function^28^, very few researches on miRNA-related SNPs in mouse have been done. Due to establishment of a number of inbred mice, isolation of several impulsive mutations has been achieved. This has enriched genetic diversity and alterations giving light to mammalian biology and human disease at large^26,27^. The fact that model animals can be kept in favorable controlled environments; inbred mice stand a better chance to give a highly reliable phenotypic data and information for genetic analysis^32^, About 3,587 mouse genotypes, modeling human diseases have been reported, with at least one or more mutated genes. Around 700 inbred mice lines have been developed and are used for genetic mapping studies^29^. The variations in these inbred lines have enabled the identification of genes and variations contributing to complex traits (QTLs) and diseases like cancers, infectious diseases^30^ physiology behavior^31^ and reproduction. Through the aid of knockout and mutant mice, a number of studies on miRNA expression comparison profiles have been achieved^38^. In a given study, significant differences on miRNA expression profiling of mice retina, showed that miRNAs are actively involved in retinal disease^39^. As a result of over expression of miR-17-92 on transgenic mice’s lymphocytes, Xiao *et al*. discovered premature death and lymphorproliferative disease in the mice^40^. miRNAs have been reported to be involved in mouse and tumor development^6^. For example in a given study several miRNAs were identified close to tumor loci in mice^41^. This clearly indicates that miRNAs processes in mice play an important role in genetics and other biological functions.

Recently, wild mice have emerged as one of the important resources for identification of genes and fine mapping. This is because, wild mice is capable of harboring larger genetic variations and have a wide historical recombination than laboratory mice^42^. Taking advantage of this capability of wild mice, Xiao *et al*., were able to develop a novel strategy fro genetic dissection of complex traits using the wild mice. as part of current research group conducted a study on complex traits using specific chromosome substitution strains of wild mice^43^. The mammalian biology has made tremendous steps due to various insights from wild mice studies^44^. In recent studies, researchers have also pointed out that wild mice has gained more preference in studying complex traits because they have been found to be easily cross bred with classical laboratory mice strains hence producing offspring with natural polymorphisms^45^. A recent study by Fuyi *et al*., also who is part of this research study team, developed an important resource for studying genetic complex traits using chromosome 1 substitution line that was a derivative of wild mice^46^. This also validates the wide and important use of wild mice in genetic research. Our current results of target gain and loss analysis showed many of the miRNAs that gained target site belong to wild mice category. This creates a big interest on these miRNAs further studies and explorations. Another study also revealed that miRNA-3473b from wild mice is directly involved in expression control of neuroinflammation in cerebral is chemia^47^. This wild type miRNA is also identified in our wild mice miRNA list having aggressive characteristics especially in controlling gene expression and can easily gain target site. In another interesting study, Tiwari *et al*., found out that mmu-mir-3473f, which belongs to wild mice category, is capable of regulating different biological processes associated with macrophage dysfunction in parasites^48^. The wild mice miRNAs seem to have a wide scoop for more analysis and various applications in genetics.

Up to date most studies on miRNA SNP analysis have been done on human and other species. We introduce the first miRNA SNP analysis with interesting results, especially our target gain and loss analysis results which showed that many of the miRNAs that gained target site belong to wild mice hence creating the arch for further studies on the same. These results outline the first case of SNP variations in the whole mouse genome, which creates a better platform for further analysis and applications on human disease.

## Materials and Methods

### Data collection

MiRNA data was retrieved from miRBase database (http://www.mirbase.org/) (Release 21)^49,50^. The SNP information was collected from the following three data sets: (1) the NCBI dbSNP146 for mouse^51^; (2) the Sanger Institute (http://www.sanger.ac.uk/science/data/mouse-genomes-project)^52^; and (3) SNPs identified on C1SLs derived from Chinese wild mice^46^.

### Identification of miRNAs related SNPs

We compared both coordinates of SNPs and the related miRNAs to identify SNPs located in mouse pre-miRNAs genes and those in their adjacent regions (flanking or 1 kb upstream/downstream regions). Intersect subcommand applied in bedtools^53^ was used to perform this analysis.

### Characterizing SNPs in mature miRNA and other sequence regions

We wrote a in home script for SNP density calculation. The assessment of SNP density differences in mature miRNAs and other sequence regions (pre-miRNA, flanking regions and up or downstream regions) was done by T test. The transition/tranversion ratio was calculated with vcftools^53,54^.

### Analysis of miRNA secondary structure

The program RNAfold implemented in ViennaRNA (version 2.2.5) package^55^ was employed to analyze miRNA secondary structure. This calculates minimum free energy (MFE), secondary structure and prints the MFE structure in bracket notation. We used RNAplot for pre-miRNAs secondary structure display^56^.

### Analysis of miRNA target loss or gain

Target gain and loss was analyzed by TargetScan (http://www.TargetScan.org/) (Release 7.2)^57^ and miRanda (http://www.microrna.org) (version 3.3a)^58^.

## Supplementary information

Dataset 1.

Dataset 2.

## Data Availability

The miRNA data used to support the findings of this study are obtained from miRBase database (http://www.mirbase.org/) (release 21) also has been cited^33^, within the article. The SNP data used to support the findings of this study was collected from the following three data sets: (1) the NCBI dbSNP for mouse (dbSNP146); (2) the Sanger Insititute (http://www.sanger.ac.uk/science/data/mouse-genomes-project) previously called SNP from whole genome resequenced 36 laboratory mice (MGPv5); and SNPs identified on chromosome 1 from 18 chromosome 1 substitution lines (C1SLs) derived from chinese wild mice as cited in the^34,35^, and^36^ respectively^59^.
